# Vibrational Spectroscopy of Homo- and Heterochiral Amino Acid Dimers: Conformational Landscapes

**DOI:** 10.3390/molecules27010038

**Published:** 2021-12-22

**Authors:** Haolu Wang, Matthias Heger, Mohamad H. Al-Jabiri, Yunjie Xu

**Affiliations:** Department of Chemistry, University of Alberta, Edmonton, Alberta, AB T6G 2G2, Canada; haolu@ualberta.ca (H.W.); heger@ualberta.ca (M.H.); aljabiri@ualberta.ca (M.H.A.-J.)

**Keywords:** chirality recognition, amino acid dimers, IRMPD spectroscopy

## Abstract

The homo- and heterochiral protonated dimers of asparagine with serine and with valine were investigated using infrared multiple-photon dissociation (IRMPD) spectroscopy. Extensive quantum-chemical calculations were used in a three-tiered strategy to screen the conformational spaces of all four dimer species. The resulting binary structures were further grouped into five different types based on their intermolecular binding topologies and subunit configurations. For each dimer species, there are eight to fourteen final conformational geometries within a 10 kJ mol^−1^ window of the global minimum structure for each species. The comparison between the experimental IRMPD spectra and the simulated harmonic IR features allowed us to clearly identify the types of structures responsible for the observation. The monomeric subunits of the observed homo- and heterochiral dimers are compared to the corresponding protonated/neutral amino acid monomers observed experimentally in previous IRMDP/rotational spectroscopic studies. Possible chirality and kinetic influences on the experimental IRMPD spectra are discussed.

## 1. Introduction

Chirality is essential for life. While a pair of enantiomers share the same chemical formula, their responses to the surrounding environment can be drastically different. While *D*-Penicillamine (Peni) is widely used as a heavy metal antagonist for its ability to bind with heavy metals such as lead [1], *L*-Peni is toxic because of its inhibition of Vitamin B6 due to their interaction [2]. Similarly, the *R*-enantiomer of thalidomide was capable of efficiently supressing morning sickness in pregnant women, whereas the *S*-enantiomer was the cause of serious birth defects, although it was discovered later that the *R*- and *S*-forms could interconvert in vivo [3,4]. The potential lethal consequence associated with chirality in pharmaceuticals has led to tighter government regulations and further development of spectroscopic tools for chirality evaluation [5]. 

Regulatory demands and the need for better chiral spectroscopic tools have also inspired considerable research efforts in characterizing chirality recognition events at the molecular level to gain a fundamental understanding of their driving forces. Chirality recognition is defined as the ability of a chiral probe, e.g., a chiral light or a chiral molecule, to differentiate between the two enantiomers of a chiral molecule [6]. While chirality recognition is well known to play an important role in biology and (supramolecular) organic syntheses, it is difficult to characterize in detail the noncovalent intermolecular interactions responsible in the condensed phase. 

In the last fifteen years or so, gas phase rotational and vibrational spectroscopies have been utilized to explore the noncovalent intermolecular interactions which lead to the preferred binding topologies for homo- and heterochiral aggregates at the molecular level. For example, Zehnacker and co-workers reported an early chirality discrimination study of a chiral naphthalene derivative with several primary and secondary alcohols using hole-burning spectroscopy [7], and more recently, together with Fujii and co-workers, using cryogenic ion trap IR spectroscopy on tyrosine and β-cyclodextrin [8]. Using jet-cooled Fourier transform IR (FTIR) spectroscopy, Suhm, Gerhards and co-workers examined aggregation behaviors of some neutral amino acids and simple peptides [9]. Jet-cooled FT microwave (FTMW) spectroscopy has been used to probe the structural diversity of some prototype systems, such as the dimers of propylene oxide [10], tetrahydro-2-furoic acid [11] and glycidol [12]. The advent of the chirped pulsed FTMW technique [13], in combination with these research results, has led to the application of chiral tag rotational spectroscopy for enantiomeric excess, ee, determination [14]. Although the extremely high resolution capability of FTMW allows one to characterize the conformational landscapes of these homo- and heterochiral complexes and differentiate structural isomers with minute differences, the size of the molecular systems possible is currently limited to about 30 heavy atoms. In this regard, infrared multiphoton dissociation (IRMPD) spectroscopy [15], coupled with the electrospray ionization (ESI) technique, offers some noticeable advantages in terms of its easy sample introduction and accessibility to larger molecular sizes.

IRMPD spectroscopy combines the detection sensitivity of mass spectrometry and the structural sensitivity of IR spectroscopy and has been extensively applied to probe the structural properties of charged amino acids and their complexes in the gas phase. For example, the binding pattern of the complexes of tryptophan with alkali cations [16] and structural diversity of di-metalized arginine [17] were researched, and Wu and McMahon examined the protonation sites and conformations of a series of amino acids [18]. Rodgers and co-workers studied the structural variation between protonated and sodiated triethyl phosphate [19], while Andersson et al. examined the structural properties of the protonated dimers of methionine and tryptophan dimers [20]. More recently, IRMPD studies of some homo- and heterochiral amino acid aggregates have also been reported. Serine is one of the most extensively investigated for its unusually stable protonated octamer clusters, originally reported by Cooks et al. [21]. Several IRMPD studies of serine clusters have been reported, to extract structural information and to provide an explanation for its “magic number” homochiral octamer preferences [22,23,24]. Related aggregates with one or more serine subunit within the octamer replaced with *S*- or *R*-threonine have also been investigated to identify any substitution effects on the preferred structural topology of the octamer [25]. A review on chirality effects in neutral and ionic complexes studied using gas phase spectroscopy was published in 2014 [26], and very recently, IRMPD chirality recognition studies were reviewed by Kong and co-workers [27], with a focus on the potential application of IRMPD spectroscopy in chiral analyses. 

In this paper, we investigated structural and energetic relationships in protonated homo- and heterochiral serine-asparagine and valine-asparagine dimers. Our interest in noncovalent interactions among amino acids in general stems from their promise as low risk co-formers for active pharmaceutical ingredients (API) which are used for improving the physiochemical properties of API [28]. More specifically, one motivation is to evaluate the ability of asparagine to differentiate a pair of enantiomers of an amino acid, an important requirement for a chiral discriminator in chiral analyses [27]. The choice of the molecular system was in part inspired by the previous report that asparagine can serve as an ee inducer in amino acid cocrystals [29]. The choice of the system was also inspired by the very different, experimental conformational distributions of neutral serine [30], valine [31] and asparagine [32] revealed by FTMW studies, where polar and nonpolar sidechains were identified as one key factor of influence. It would be of interest to examine what role the polar or nonpolar sidechains play in the respective dimer structures, and also how noncovalent interactions influence the conformational distributions of the neutral/charged amino acid subunits. 

## 2. Results and Discussion

**Scheme 1:**
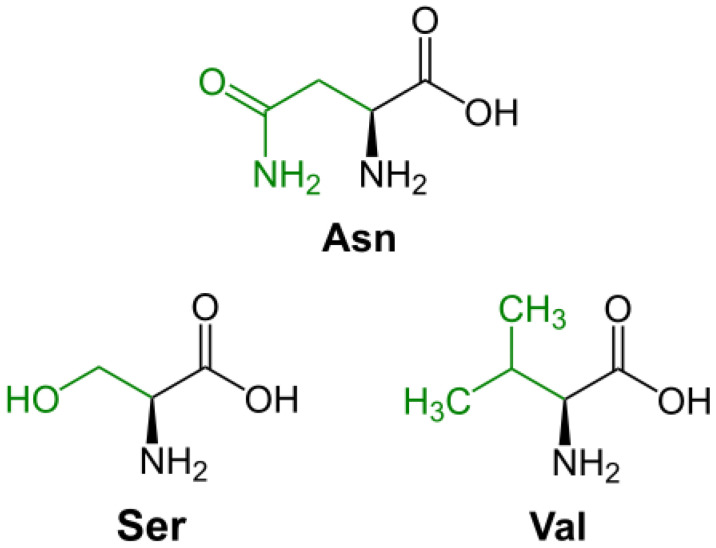
Structural formulas of the three neutral amino acids used in the current study. The sidechains are highlighted in green for asparagine (Asn, **top**), serine (Ser, **bottom left**) and valine (Val, **bottom right**). All amino acid monomers shown here are the *S* enantiomers.

The structural formulas of all three neutral amino acid monomers are shown in Scheme 1. Four homo- and heterochiral binary adducts were studied, namely *S*,*S*-HSerAsn^+^, *R*,*S*-HSerAsn^+^, *S*,*S*-HValAsn^+^ and *R*,*S*-HValAsn^+^, all of them with *S*-asparagine. The corresponding four mirror images with *R*-asparagine would give rise to the same IR spectra; thus, only the binary adducts containing *S*-asparagine are used in the remainder of the paper. 

### 2.1. Possible Low Energy Structures of the Four Diastereomers

While chemical intuition and prior experimental structural evidence have been used in some reports, such as in the IR spectroscopic study of (Ser_8_Cl_2_)^2−^ [33] and (HSer_8_)^+^ [23], increasingly a global search for suitable structures has been carried out by the laser-mass spectrometry community using some MD simulation program packages, such as Macromodel by Schrödinger [34] and DFTB+, which utilizes the DFT-based tight binding method [35,36]. For example, Poline et al. applied this approach to investigate the IR signature of several homo- and heterochiral protonated amino acid dimers [37]. We chose to use the conformer-rotamer ensemble sampling tool (CREST), developed by Grimme and co-workers [38]. CREST has been applied extensively and successfully to rotational spectroscopic studies of the conformational landscapes of mid-sized neutral organic molecules and their clusters [39,40]. Furthermore, it was also benchmarked for its ability to correctly predict protonation sites [41].

In some recent rotational spectroscopic studies of fluoroalcohol trimers and tetramers [42,43], it was recognized that monomeric conformations which are not stable in their isolated form, may become the main or even the only subunits in larger aggregates, highlighting the importance of extensive sampling of the conformational space. While each CREST run already has 12 built-in MD runs, to cover as much conformational space as possible, multiple CREST runs with the same or different starting geometries were added in the current study. One reason is that while performing conformational analyses, we noticed that a high torsional barrier exists in all species, which locks the OH group of the carboxylic group in either a cis or trans configuration relative to the C=O group. A similar phenomenon was reported in the rotational spectroscopic studies of tetrahydro-2-furoic acid [44] and its dimer [11]. In a single CREST run, redundant structures are removed by the program itself. Since many conformers found in different CREST runs could also be the same, we used a Python program to calculate the root-mean-square deviation (RMSD) of atomic Cartesian coordinates for the obtained conformations. A RMSD value of zero indicates identical conformations, whereas the larger the value, the more unalike the two conformations are. By trial and error, a RMSD threshold of 0.6 was used. 

While some have chosen to directly use the xTB energy to choose structural candidates for further DFT optimization, our experience with neutral aggregates suggested that the xTB energy ranking may be misleading in some cases [40]. An initial survey of the single-point energies of the CREST geometries was performed and the result suggested that such energy values were still too far from the final ones for them to be used as credible discriminators. Because of the large number of initial CREST candidates, we decided to add a few additional fast computational steps to obtain a more reliable energy ranking so that we could properly select the low energy structures for final geometry optimization and harmonic frequency calculations. These include (1) CREST output; (2) DFT optimizations with a relaxed convergence criteria of initial CREST candidates at the revPBE-D3/def2-SVP [45] level, with the empirical D3 dispersion correction [46,47]; and (3) a single-point energy evaluation at the B3LYP-D3/def2-TZVP level of the optimized structures in step (2). 

**Figure 1:**
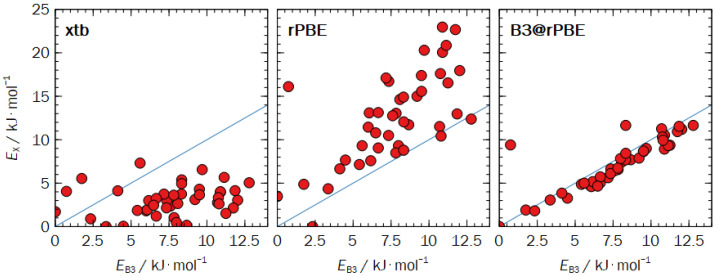
Correlation between 42 calculated *S*,*S*-HSerAsn^+^ energies, E_x_, at various levels of computation with the B3LYP-D3/def2-TZVP energies, E_B3_. The blue line indicates that E_x_ = E_B3_. From left to right: the GFN2-xTB energies from the CREST (‘xtb’); revPBE-D3/def2-TZVP after geometry optimization (‘rPBE’); and B3LYP-D3/def2-TZVP single-point calculations of the revPBE-optimized structures (“B3@rPBE”).

Figure 1 shows the relative energies for 42 *S*,*S*-HSerAsn^+^ structures at the three different calculation stages and their correlation with the B3LYP-D3/def2-TZVP energies after geometry optimization using Molpro [48,49]. It is apparent that GFN2-xTB generally underestimates the relative energies of the CREST conformers, resulting in “overcrowding” of the initial conformer ensemble within a given energy window, making it difficult to carry out the selection. The revPBE relative energies, on the other hand, tend to be overestimated. Finally, single-point B3LYP calculations of the revPBE-optimized structures yield very reliable estimates of the B3LYP-D3/def2-TZVP energies after geometry optimization, save for a few outliers. This combined B3LYP-D3/revPBE-D3 step is therefore crucial in assessing the conformer ensembles, since it strikes a very attractive balance between computational effort and accuracy without requiring the full B3LYP-D3 optimizations.

For the four chiral diastereomers of interest, the final optimization and frequency calculations of the structures selected within an energy window of ~10 kJ mol^−1^ from the global minimum of each species were performed at the B3LYP-D3BJ/def2-TZVP level using Gaussian 16 [50]. The energetic properties and the associated Boltzmann factors are summarized in Tables S1–S4 (Supplementary Material) for *S*,*S*-HSerAsn^+^, *R*,*S*-HSerAsn^+^, *S*,*S*-HValAsn^+^, and *R*,*S*-HValAsn^+^, respectively. The single-point energy calculations of all the above structures with the inclusion of a solvent polarizable continuum model (PCM) implemented in Gaussian 16 [50] are also included in the corresponding Tables. Unsurprisingly, the zwitterionic (ZW) form of these binary clusters is strongly preferred with the inclusion of a solvent.

**Figure 2:**
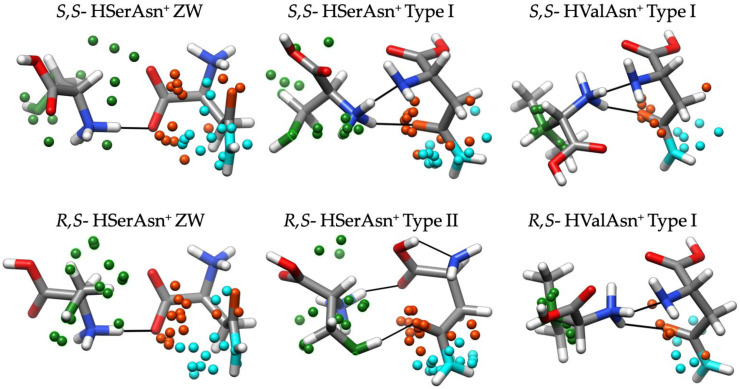
Global minimum structures of the Type ZW, Type I and Type II binary species. See Section 2.2 for the definition of different types. The O and N atoms in the Asn sidechain are coloured in orange and turquoise, respectively, and the O and tertiary C atoms in the Ser and Val sidechains are coloured in green. The additional points indicate the positions of the highlighted sidechain atoms among all other final structures.

The global minimum structures of each species are presented in Figure 2, as well as the second most stable structures of *S*,*S*-HSerAsn^+^ and *R*,*S*-HSerAsn^+^. In addition, to visualize the full range of sidechain orientations that each dimer species realizes within the 10 kJ mol^−1^ window, important atoms are color coded. The matching dots are then used to indicate the positions of these specific atoms in all structures beyond the explicitly depicted minima after alignment by RMSD minimization of all non-hydrogen atoms. All these structures have the protonation site on Ser or Val, while Asn takes on the neutral or ZW form.

### 2.2. Different Noncovalent Binding Topologies

In the previous rotational spectroscopic studies of the conformational landscapes of monomeric, neutral amino acids [51], it was recognized that α-amino acids with a nonpolar sidechain such as Val, typically present in only two dominant conformers stabilized by either a bifurcated N–H⋯O=C hydrogen bond with a cis-COOH configuration or a N⋯H–O hydrogen bond [31]. With the presence of polar sidechains, the number of conformers with similar energies tends to increase dramatically. For example, seven conformers were observed for neutral Ser [30]. Interestingly only one main conformer of Asn was identified in the previous rotational spectroscopic study [32], an exception to the rule. Asn utilizes both cis- and trans-COOH configurations in the binary species studied here, although it can take on neutral, protonated, or ZW forms, a point which will be further discussed in Section 2.4. Generally, the greater conformational diversities of the Ser monomer versus Val also seem to be reflected in their respective protonated binary species (Figure 2), where the sidechain of the Ser subunit has the tendency to occupy far more different regions in the above dimers than the sidechain of Val.

To better appreciate structural diversities in the homo- and heterochiral HSerAsn^+^ and HValAsn^+^ dimers, we divided these isomers into five types based mainly on their key intermolecular interaction difference, the subunit conformations and whether the subunits take on the ZW form or not. These include Type I, II, and III of the protonated form where the IR band signatures look very similar within each type but different from each other, then the ZW form, and finally some minor structures labelled as ‘Other’ which do not belong to the previous four categories. These labels are also listed in Tables S1–S4 for all four binary species. Based on the predicted relative free energies, Type I, II, and III of HValAsn^+^ make up almost all the population, whereas those labelled as ‘Other’ contribute very little. In contrast, for HSerAsn^+^, those labelled as “Other” have a higher contribution. This outcome is expected since the polar sidechain of Ser offers more potential binding sites with Asn.

**Figure 3:**
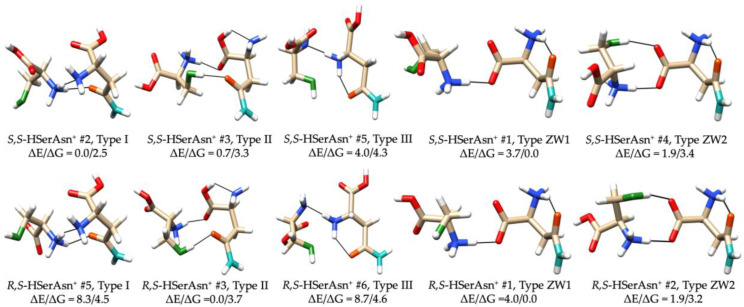
Type I, II, III, and ZW binding topologies of *S*,*S*-HSerAsn^+^ and *R*,*S*-HSerAsn^+^. The geometries shown are the most stable isomers in each type. The monomer subunit on the left is S- or R-Ser, while the one on the right is S-Asn. The relative ZPE corrected electronic energy (ΔE) and Gibbs free energy (ΔG) at room temperature in kJ mol^−1^ at the B3LYP-D3BJ/def2-TZVP level of theory are included. The ranking #1, #2, etc., is based on ΔG and is done for the homo- and heterochiral HSerAsn^+^, separately. The ΔE and ΔG values between the most stable isomers of *S*,*S*- and *R*,*S*-HSerAsn^+^ are 0.4 and 0.4 kJ mol^−1^, respectively, with *S*,*S*-HSerAsn^+^ Type ZW1 #1 being the most stable one.

In Figure 3, the geometries of the most stable isomers of each type of *S*,*S*-HSerAsn^+^ and *R*,*S*-HSerAsn^+^ are provided, where the dominant intermolecular hydrogen bonds are also indicated, together with their relative energies. Since the relative stability ranking of the isomers might change based on ΔE or ΔG, we used ΔG for ranking in the remainder of the paper. Type I structures contain two intermolecular hydrogen bonds connecting the NH_3_^+^ group of Ser with the nitrogen atom and the carbonyl oxygen atom of Asn. Type II isomers have an intermolecular hydrogen bond between the Ser NH_3_^+^ group and the carboxyl O atom of Asn and another from the Ser OH group to the carbonyl O atom of Asn. At the same time, the carboxyl OH group of Asn maintains an intramolecular hydrogen bond with its own nitrogen atom where the backbone dihedral angle of the Asn subunit needs to be turned to facilitate this intramolecular hydrogen bond, leading to a slight destabilization of the whole structure. Type III isomers are unique compared to the other three because the protonation site is on the Asn subunit instead of Ser. In Type III isomers, the nitrogen atom of Ser forms an intermolecular hydrogen bond with the NH_3_^+^ group of Asn, while an intramolecular hydrogen bond is formed between the carbonyl O atom of Ser and its NH_2_ group. As the Asn subunit takes on the ZW form, two binding motifs are identified for HSerAsn^+^: ZW1 where the polar sidechain of Ser is not involved in the intermolecular interaction and ZW2 where the Ser sidechain is involved. By changing how a subunit approached the H atom(s) of the NH_3_^+^ group of Ser to form the respective hydrogen bonding interactions, three isomers having Type I structures were found, and two for each of Type II, III and ZW2. 

**Figure 4:**
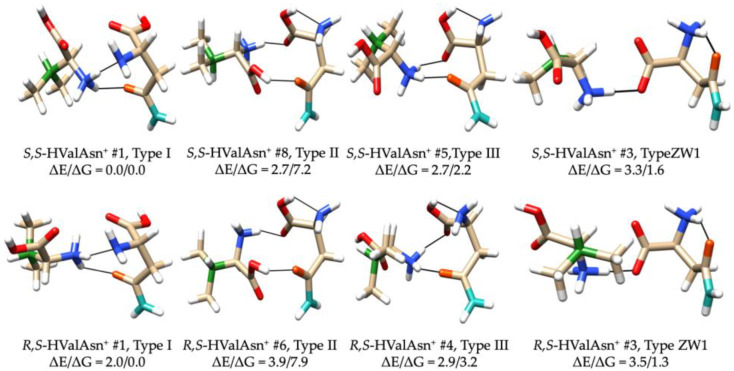
Type I, II, III, and ZW binding topologies of *S*,*S*-HValAsn^+^ and *R*,*S*-HvalAsn^+^. The geometries shown are the most stable isomer in each type. The monomer subunit on the left is *S*- or *R*-Val, while the one on the right is *S*-Asn. The relative ZPE corrected electronic energy (ΔE) and Gibbs free energy (ΔG) at room temperature in kJ mol^−1^ at the B3LYP-D3BJ/def2-TZVP level of theory are included. The ranking #1, #2, etc., is based on ΔG and is done for the homo- and heterochiral HvalAsn^+^, separately. The ΔE and ΔG values between the most stable isomers of *S*,*S*- and *R*,*S*-HvalAsn^+^ are 1.7 and 0.7 kJ mol^−1^, respectively, with *S*,*S*-HvalAsn^+^ Type I #1 being the most stable one.

Similarly, the four types of binding topologies of *S*,*S*-HValAsn^+^ and *R*,*S*-HValAsn^+^ are depicted in Figure 4. Type I and II structures of the HValAsn^+^ species contain very similar intermolecular hydrogen bonds as in the corresponding Type I and II of the HSerAsn^+^ species, respectively. The exception is that in the Type II of the HValAsn^+^ species, the COOH of Val acts as a proton donor instead of the OH group of Ser in the HSerAsn^+^ species case. This is not surprising since Val does not have an alcohol OH group. Type III of the HValAsn^+^ species utilizes the NH_3_^+^ of Val as the proton donor to the carbonyl and carboxylic O atoms of Asn, a very different binding topology compared to that of the HSerAsn^+^ species. Unlike HSerAsn^+^, only one binding motif is observed for the ZW structures since their nonpolar sidechain is not a competitive intermolecular hydrogen bond donor candidate compared to the OH group of Ser.

**Figure 5:**
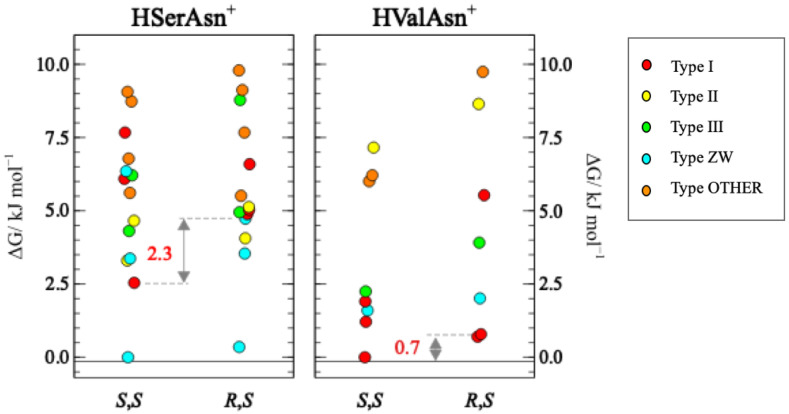
The relative free energies of the four dimer species. The energy gap between the experimentally observed homo- and heterochiral dimers of HSerAsn^+^ is 2.3 kJ mol^−1^ and that of HValAsn^+^ is 0.7 kJ mol^−1^.

Figure 5 visualizes the relative free energies of all the final structures within the 10 kJ mol^−1^ window for the four dimer species. The data points in each trace are coloured according to their binding topologies: Type I, II, III, ZW and ‘Other’. In the case of the HserAsn^+^ species, the lowest energy structures of the homo- and heterochiral complexes appear to be the same type, Type ZW. For the HvalAsn^+^ species, the lowest energy structures of the homo- and heterochiral complexes also appear to be the same type, Type I.

### 2.3. Comparison of the Experimental and Theoretical IRMPD Spectra

**Figure 6:**
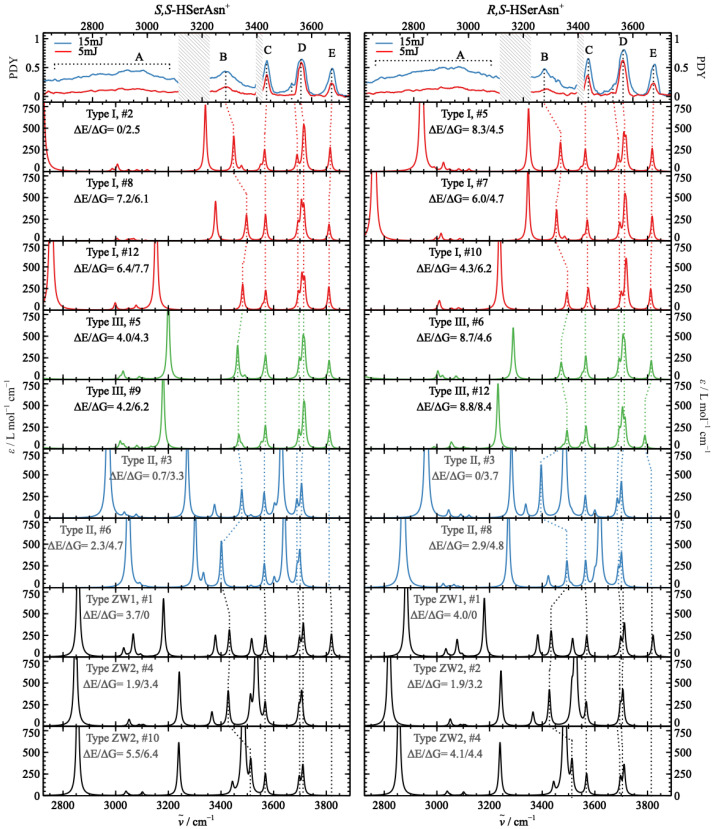
Top panel: experimental IRMPD spectra of *S*,*S*- and *R*,*S*-HSerAsn^+^ dimers under two laser energies. The two laser “blind” regions mentioned in the experimental section are indicated by the shaded areas. Lower panels: theoretical IR spectra of the most stable isomers of Type I (red), Type II (blue), Type III (green), and ZW (black). The relative electronic energy (ΔE) and Gibbs free energy (ΔG) values of the respective isomers are given in units of kJ mol^−1^. The theoretical IR bands correspond to the same (similar) vibrational motions and are connected with dashed lines which in turn connect to the experimental bands to indicate the assignment.

In Figure 6, the experimental IRMPD spectra of the homo- and heterochiral HSerAsn^+^ dimers are compared with the theoretical, individual IR spectrums of the most stable isomers of the five relevant types, while detailed comparisons between the experimental and theoretical spectra of each type are summarized in Figures S1 and S2, Supplementary Material. Both homo- and heterochiral experimental spectra show four clear band features, labelled as B, C, D, and E, in the above 3200 cm^−1^ region. Experimentally, there is also a small shoulder band to the lower wavenumber side of D which only becomes obvious at a higher radiation energy of 15 mJ. Based on the comparison between the experimental and simulated spectral features, one can rule out contributions of Type II, ZW1 and ZW2 for both *S*,*S*-HSerAsn^+^ and *R*,*S*-HSerAsn^+^. Below, we focus on the remaining Type I and III structures.

For *S*,*S*-HSerAsn^+^, Type I, #2 provides the best overall agreement with the experimental IR pattern including the small shoulder band next to D and in terms of the predicted energetic preference, i.e., the most stable one among the remaining Type I and III. The other four isomers, i.e., Type I, #8 and #12, and Type III, #5 and #9, all show features consistent with C, D, and E. It appears that the contribution from them can be used to explain the broadening observed in the experimental D band, and also the much broader B band where the corresponding calculated B bands of these isomers extend over some tens of wavenumbers. 

In terms of the vibrational assignment, the highest frequency E band corresponds to the free OH stretch of Ser which is not involved in the noncovalent intermolecular interaction; the D band can be related to the carboxyl OH stretches of the Ser and Asn subunits. Neither of the carboxyl OH stretches are involved in the intermolecular hydrogen bonds and are predicted to be close in their frequencies. The “shoulder” peak (next to D) at ~3550 cm^−1^ corresponds to the asymmetric stretching motions of the sidechain NH_2_ group of Asn. The C band contains the symmetric stretching information of the sidechain NH_2_ group of Asn, as well as the asymmetric stretching motion of the proton acceptor NH_2_ group of Asn, the intensity of which is lower and appears as a smaller “shoulder” in the predicted spectra. The B band is assigned to a collection of symmetric stretch motions of the proton acceptor NH_2_ group of Asn and the asymmetric NH_2_ vibrations in the proton bound NH_3_^+^ groups of Ser, analogous to the previously published assignments [24]. Note that the predicted symmetric NH_2_ vibrations in the NH_3_^+^ group of Ser fell into the “blind region” of our laser and could not be detected in the experiment.

In the region below 3200 cm^−1^, the predicted IR bands are dominated by the stretching modes of NH_3_^+^ which serves as a hydrogen bond donor in the dimers. These stretching bands exhibit typical characters such as large red shifts and a big enhancement in IR strength. Experimentally, it is well known in the jet-cooled high-resolution IR community that IR photons pumped into intermolecular hydrogen bonds tend to lead to severe predissociation broadening in the experimental IR spectra [52]. In the current case, this results in a broad and featureless contour which is marked as band A, similar that which was observed previously [24]. The backbone CH stretches from the Asn and Ser subunits are predicted to be in the 3000–3110 cm^−1^ region and tend to be featureless because of the high density of the CH vibrational states [22]. 

For *R*,*S*-HSerAsn^+^, the Type I, #5 and #7 isomers appear to provide the best agreement with the experimental observations, while the contribution from Type I, #10 and Type II, #6 and #12 is also present. The IR band assignments for A–E remain analogous to those of *S*,*S*-HSerAsn^+^. There are some minor experimental pattern differences from *S*,*S*-HSerAsn^+^: the D shoulder is less well resolved and some partially resolved structures in B are more obvious. These experimental observations can be explained by the smaller separation predicted for the D band and its shoulder band in Type I and III in *R*,*S*-HSerAsn^+^ and because the free energy gaps among the Type I and III isomers are smaller than the gap between Type I, #2 and the rest in the case of *S*,*S*-HSerAsn^+^, leading to a wider contribution of different isomers.

**Figure 7:**
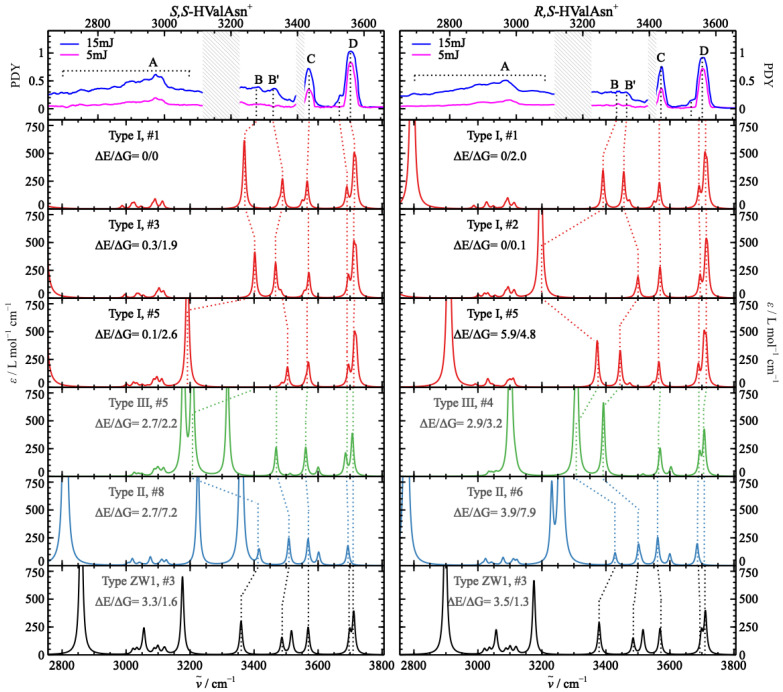
Top panel: experimental IRMPD spectra of *S*,*S*- and *R*,*S*-HValAsn^+^ dimers under two laser energies. The two laser “blind” regions mentioned in the experimental section are indicated by the shaded areas. Lower panels: theoretical IR spectra of the most stable isomers of Type I (red), Type II (blue), Type III (green), and ZW (black). The relative electronic energy (ΔE) and Gibbs free energy (ΔG) values of respective isomers are given in units of kJ mol^−1^. The theoretical IR bands correspond to the same (similar) vibrational motions and are connected with dashed lines which in turn connect to the experimental bands to indicate the assignment.

In Figure 7, the experimental IRMPD spectra of homo- and heterochiral HValAsn^+^ dimers are compared with the simulated individual IR spectrums of the most stable isomers of Type I, II, III and ZW1, while detailed comparisons between the experimental and theoretical spectra of each type are summarized in Figures S3 and S4, Supplementary Material. Type ZW2 is outside the 10 kJ mol^−1^ free energy window. 

The most obvious difference from the HSerAsn^+^ case, is the missing E band which belongs to the free OH of Ser, since HValAsn^+^ does not have such a free OH group. Based on the simulated IR spectral patterns in the region surrounding C, we can rule out the contribution of Type II and III. Both of them have a lower wavenumber band predicted next to C, which is not present in the experiment. The comparison of the simulated and experimental band gap between C and B’/B also allows one to discard the contribution of Type ZW1. 

For *S*,*S*-HValAsn^+^, Type I, #1, #3 and #5 structures exhibit well-aligned C and D bands, i.e., very similar C and D frequencies among the three isomers, and more spread out B′/B bands, consistent with the experimental spectral appearance. The B and B’ of the Type I structures are assigned to the symmetric and asymmetric stretches of the hydrogen bonded NH_3_^+^ group of Val. The IR assignments of the C and D features are analogous to the HSerAsn^+^ case described above.

Similarly, for *R*,*S*-HValAsn^+^, Type I, #1, #2, and #5 structures contribute to the experimental IR spectra, as indicated by the good agreement with the experimental features. Contributions of Type II, Type III and Type ZW1 structures can be discarded for the same reasons provided above for *S*,*S*-HValAsn^+^.

### 2.4. Chirality Recognition and Kinetic Effects in the IRMPD Spectra

The above IRMPD spectral analyses show that while one could clearly discriminate among different types of structures for the four binary species studied here, only minor differences between the homo- and heterochiral species of HSerAsn^+^ and HValAsn^+^ were observed experimentally. Does this mean there is no or little chirality recognition in these homo- versus heterochiral species? In the following, we first address why the species which contribute to the experimental IRMPD patterns may not necessarily be the most stable structures predicted. Then we discuss some noticeable chirality recognition signatures and energetic differences in these systems, even if these are not reflected in the IRMPD features detected, and how one may tease out these signatures with modified experimental approaches. 

Although the global minima predicted for both *S*,*S*-HSerAsn^+^ and *R*,*S*-HSerAsn^+^ belong to the ZW type, they were not observed experimentally. With respect to the most stable non-ZW heterochiral and homochiral dimer structures, *R*,*S*-HSerAsn^+^ Type II, #3 has a drastically different IR pattern compared to that of *S*,*S*-HSerAsn^+^ Type I, #2. This prediction appears to *contradict* the previous statement, that experimentally only minor differences are present between the homo- and heterochiral IRMPD spectra (Figure 6). Furthermore, the observed heterochiral IRMPD spectra of HSerAsn^+^ can be accounted for mainly by *R*,*S*-HSerAsn^+^ Type I structures, with essentially no contribution from the *R*,*S*-HSerAsn^+^ Type II, #3 isomer, the most stable non-ZW form of *R*,*S*-HSerAsn^+^ predicted. While the predicted energy ordering or gaps may not be totally trustworthy, the level of theory used here has generally captured the energy ordering of similar neutral species quite well, as demonstrated by many examples reported by the rotational spectroscopic community [53,54]. On the other hand, kinetically trapped neutral species in a jet expansion [11] and in electrosprayed ions [55] have been reported before. To explain the observation discussed above, we also examine if the amino acid dimers are mainly formed in solution or in the gas phase during the electrospray processes and the influence of the relative stabilities (i.e., abundances) of the monomeric subunits. 

In Table 1, we list the monomeric composition of the most stable isomers of each type for HSerAsn^+^ and HValAsn^+^, while the related results of all low energy binary isomers are provided in Table S5. In a previous study, Zhu et al. [56] estimated the degree of self-aggregation of Ser in water with respect to the concentration. Eight different concentrations, ranging from 0.1 M to saturation were studied, and no severe self-aggregation was observed in any of them. The concentration of our sample solution is ~3 mM, much lower than the concentrations used in the previous study, indicating that the formation of amino acid dimers in the millimolar solution is likely to be negligibly small. HSerAsn^+^ and HValAsn^+^ are probably formed mainly during the electrospray process. While amino acids exist mainly as zwitterions in a pH (near) neutral aqueous solution, an isolated amino acid exists dominantly in a non-ZW form in the gas phase. This is the case even for the most basic amino acid, arginine [57]. The poor stability of the zwitterionic form in the gas phase provides an explanation for the non-observation of any binary species with zwitterionic subunits, irrespective of their predicted relative free energies. 

The homo- and heterochiral HSerAsn^+^ and HValAsn^+^ which have been assigned in the experimental IRMPD spectra are shaded in Table 1. It is interesting to note that all of them contain the most stable monomeric protonated species: HS1^+^, HV1^+^ and HA^+^. We note that HS1^+^ identified here is also the most stable isomer reported in the previous IRMPD studies by Wu et al. [18] and Sunahori et al. [24]. HV1^+^ corresponds to the most stable conformation reported for the protonated monomer of valine methyl esters [58]. Two similar HA^+^ configurations are utilized in the dimers and correspond to the two most stable protonated Asn isomers reported by Heaton and co-workers [59] and by Heger et al. [60], with their structures differing slightly depending on which carbonyl O lone pair is used in the intramolecular H-bond with the NH_3_^+^ group.

The connection to the stability of the neutral monomeric species is less clear. For example, the observed Type I *S*,*S*-HSerAsn^+^ is made of HS1^+^/A1, where A1 (*cis*-COOH) has a very similar configuration to Ic, a higher energy isomer, reported in a previous jet microwave spectroscopic study [32]. The non-observed Type III *S*,*S*-HSerAsn^+^, on the other hand, is made of HS1^+^/A2, where A2 (*trans*-COOH) takes on a structure somewhere between II_a_, the only one observed experimentally in a jet, and II_b_, a much higher energy isomer [32]. The observed Type III structure of HSerAsn^+^ is made of S1/A^+^, where S1 has a structure somewhere between I_b_ and I′_b_, two higher isomers of Ser [30]. These observations are not too surprising because in a protonated dimer, the neutral subunit often opens up some of its intramolecular hydrogen bonds to accommodate a strong intermolecular interaction with its protonated counterpart. It is noted that structural interconversions, for example, the proton migration which is discussed in Section 2.5, may complicate the discussion of the formation of gas-phase ions, although one would expect such processes to lead to more stable species. Overall, the abundances of the monomeric ZW and protonated subunits in the gas phase seems to play an important role in which dimer can be observed experimentally, rather than just the relative thermodynamic stability of the dimers. 

To explore the possibility that chirality recognition effects may be detected in some other frequency regions, the predicted IR spectra in the 0–2650 cm^−1^ region of all the *assigned* structures, namely Type I and Type III structures of HSerAsn^+^ and Type I structures of HValAsn^+^ are depicted in Figure S5, Supplementary Material. The zoom-in spectra in the 1000–1900 cm^−1^ region are also shown. The 1600–1700 cm^−1^ region offers potentially the most noticeably different IR band features which are associated with the NH_x_ scissoring of the protonated NH_3_^+^ and sidechain NH_2_ functional groups, as well as the NH_3_ umbrella bending motion.

Very recently, Andersson et al. reported minor differences in the IRMPD spectra of the homo- and heterochiral proton-bound Asn dimer [61], similar to what we observe in terms of the chirality effects in the IRMPD spectra of the four species discussed. Based on their experiment and also a related theoretical study [37], the authors suggested that to observe chiral differences within the mid-IR region, a sidechain must be involved in the intermolecular interactions. This hypothesis also appears to apply in the simulated IR spectra of several types of the current species. For example, Type II *S*,*S*- and *R*,*S*-HSerAsn^+^ isomers have *both* sidechains of Ser and Asn involved in intermolecular interactions, and noticeable chiral effects, i.e., differences in the homo versus heterochiral IR spectra, are predicted. On the other hand, Type III structures have the least sidechain involvement in the intermolecular interactions and their homo- and heterochiral IR spectra are more similar, showing almost no chiral effects. For the protonated Asn dimers, the authors also suggested that dimers with limited interactions with the sidechain are energetically favored. This does not appear to apply to the current systems since the most favored binary species (based on the theoretical and experimental results) are Type I structures which have more or similar sidechain involvement as compared to the other Types of structures.

**Figure 8:**
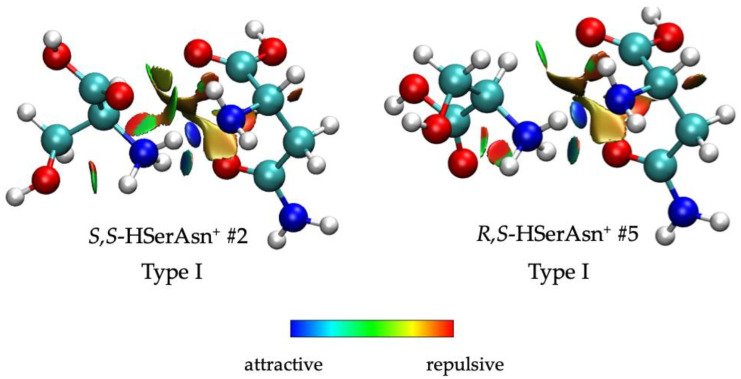
NCI plots of the most stable homo- and heterochiral HSerAsn^+^ at an iso-surface value of 0.5.

It is interesting to point out that the ZW types of HSerAsn^+^ and HValAsn^+^ are predicted to have a slight homochiral preference (see Figure 3 and Figure 4), consistent with the homochiral preference trend reported in Ref. [29], where in a racemic mixture of Ser or Val with an excess of *R*-Asn, *R*-Ser or *R*-Val preferentially co-crystallized, respectively. Since none of these ZW types were observed in the current experimental study, to appreciate the role that chirality plays in the energy ordering, we carried out noncovalent interaction (NCI) analyses [62] and quantum theory of atoms in molecules (QTAIM) [63] analyses of the experimentally observed Type I binary homo- and heterochiral HSerAsn^+^ species. The NCI analyses are depicted in Figure 8. As one can see in Figure 8, the intermolecular hydrogen bonds are from the NH_3_^+^ group of Ser to the NH_2_ and the O of the carbonyl group of Asn in both homo- and heterochiral dimers, whereas Ser has its carboxyl and the hydroxyl groups pointing away from Asn. The NCI plots also show other intermolecular interactions besides the two hydrogen bonds mentioned above, more for *S*,*S*- than *R*,*S*-HSerAsn^+^. To quantify the strength of the main hydrogen bonds, we also carried out a QTAIM analysis and used the recently derived equation for charged complexes to estimate the associated intermolecular interaction bond energies [64]. The bond energies of the intermolecular N–H^+^⋯O interactions for the homo- and heterochiral HSerAsn^+^ are 44.7 kJ mol^−1^ and 41.7 kJ mol^−1^, respectively, very similar in strength. In contrast, the bond energies of the N–H^+^⋯N intermolecular hydrogen bonds for the homo- and heterochiral HSerAsn^+^ are 68.2 and 57.5 kJ mol^−1^, respectively, ~11 kJ mol^−1^ smaller for *R*,*S*-HSerAsn^+^ than for *S*,*S*-HserAsn^+^. It appears that constrained by its chirality, it is more difficult for *R*,*S*-HserAsn^+^ to optimize its intermolecular interactions while it sustains/minimizes the attractive/repulsive intramolecular interactions simultaneously, than *S*,*S*-HserAsn^+^. Overall, a noticeable chirality recognition energy gap of 8.3 kJ mol^−1^ (ZPE corrected energy) and 2.3 kJ mol^−1^ (free energy) respectively, is present for *R*,*S*- versus *S*,*S*-HserAsn^+^, with the latter being more stable. 

In the case of Type I *S*,*S*- and *R*,*S*-HvalAsn^+^, the NCI plots are provided in Figure S6, Supplementary Material. The corresponding QTAIM intermolecular N–H^+^⋯O bond energies are 42.5 and 35.3 kJ mol^−1^ for the Type I *S*,*S*- and *R*,*S*-HValAsn^+^, respectively. For the N–H^+^⋯N bond, these values are 70.3 and 65.3 kJ mol^−1^ for the Type I *S*,*S*- and *R*,*S*-HValAsn^+^, respectively. Overall, the chirality recognition energy is about only 1.7 kJ mL^−1^ (ZPE corrected energy) and 0.7 kJ mol^−1^ (free energy) in favour of the homochiral dimer. A plausible explanation is that Val has a nonpolar sidechain which is less involved in noncovalent interactions, leading to less influence on chirality recognition. As a result, the chirality recognition energy of this system is relatively small by comparison to that of HSerAsn^+^.

In comparison to the typical chirality recognition energies encountered in the neutral homo- and heterochiral dimers, for example in those containing transient chiral subunits investigated using jet-cooled FTMW spectroscopy [65,66], the values predicted for the current series of homo- and heterochiral dimers are much larger. Certainly, the very high spectral resolution associated with jet-cooled FTMW spectroscopy provides a significant advantage in resolving possible conformers, over the current IRMPD spectroscopy. If one can lower the experimental temperature to, for example 100 K, one would reduce the number of populating isomers and obtain a less crowded spectrum. At 100 K, one would expect only Type I, #2 for *S*,*S*-HSerAsn^+^, and Type I, #7 for *R*,*S*-HSerAsn^+^ based on both the thermodynamic and kinetic controlled processes discussed, leading to more obvious differences between the homo- and heterochiral IR spectra (Figure 6). Indeed, a recent low temperature study of protonated glutamic acid dimers demonstrated that a cryogenic temperature approach could offer more clarity on chirality recognition spectral signatures [67]. 

### 2.5. Fragmentation Channel

For all four species, their fragment mass spectra were strongly dominated by the protonated HAsn^+^ monomer at *m*/*z* 133, with minor contributions from smaller fragments at *m*/*z* 87 and 115 which were observed at specific irradiation wavenumbers. The observation is consistent with the expectation that these dimers can photodissociate into intact monomer units easily since their amino acid subunits are bound via weak noncovalent interactions, while any further “secondary” fragmentation of the monomers would require higher energies to overcome the dissociation threshold of covalent bonds. It is interesting to note that while the protonated HAsn^+^ monomer peak at the *m*/*z* of 133 dominates the spectra, there is no sign of the corresponding HSer^+^ or HVal^+^ peaks at the *m*/*z* of 106 and 118. We further verified if HSer^+^ or HVal^+^ experienced secondary fragmentation into even smaller pieces and found only smaller fragments at *m*/*z* 87 and 115 which could be attributed to the secondary fragmentation of HAsn^+^ [60,68].

The detection of the HAsn^+^ fragment ions exclusively for all four species was somewhat unexpected initially. The calculations suggest that the intact dimer structures generally prefer protonation on the Ser or Val units (cf. Section 2.2. *Different Noncovalent Binding Topologies*), with the exception of Type III HSerAsn^+^, where the proton prefers to stay with the Asn subunit instead of Ser. It is possible that the excess proton may migrate efficiently in the Type I structures from the Ser/Val amino groups to the Asn molecule during the photodissociation process. Two requirements are needed to achieve the above outcome: (1) the HAsn^+^ fragment must be more stable than HSer^+^ or HVal^+^ and (2) the required proton migration must happen with nearly complete efficiency during the dissociation process. The thermodynamic aspect can be explained on the grounds that the proton affinity (PA) of Asn (~940 kJ⋅mol^−1^) is higher than Val (~910 kJ⋅mol^−1^) and Ser (~920 kJ⋅mol^−1^) [69,70]. In terms of the kinetic aspect of the proton migration, since the fragmentation process is highly energetic [71], the proton may become loosely bonded between the two monomer subunits, largely free to migrate from one subunit to another. One can draw some comparison to proton mobility effects previously observed by Hopkins et al. in dimers of 3-cyanophenylalanine and trimethylamine, where irradiation of different vibrational bands in the fingerprint region led to different proton transfer patterns between the two dissociating fragments [72]. 

## 3. Materials and Methods

### 3.1. Sample Preparation

Both *SS* and *RS* sample solutions for the ESI production of HSerAsn^+^ and HValAsn^+^ were prepared using purified water and methanol as solvents at a volume ratio of 50:50. The Asn, Ser, and Val samples (Sigma-Aldrich, St. Louis, MO, USA) were used as received without further purification. 

### 3.2. IRMPD Experimental Details 

The experiments were carried out using a recently completed IRMPD mass spectrometer, “SCORPION” [67]. Since the previous report [60], its completion, extensive modifications have been made and these changes are provided in Point S1, Supplementary Material. Briefly, the spectrometer consists of an electrospray ionization (ESI) source, a quadrupole mass filter, and two separate “arms”: one with a 3D Paul ion trap for IRMPD experiments while the other has a linear quadrupole ion trap and is coupled to an existing helium nanodroplet isolation (HENDI) instrument [73,74]. For the IRMPD experiments, we used a reflectron time-of-flight detector for the acquisition of the mass spectra. Two tunable, continuous wave OPO lasers (Lockheed-Martin Argos Aculight, modules B and C) were used as the IRMPD light source. Module B has two “blind regions” from 3400 to 3420 cm^−1^ and 3120 to 3220 cm^−1^. A step size of 2 to 5 cm^−1^ and two nominal laser irradiation energies of 5 mJ and 15 mJ were used. The latter was achieved by installing a remote-controlled laser shutter in the laser beam path and controlling the irradiation timing to achieve the “nominal” irradiation energy (laser power multiplied with shutter opening time) during each measurement cycle. In the current setup, we recorded alternating “irradiated” and “nonirradiated” (background) mass spectra of the trapped ion samples, thus correcting for self-dissociation of the targeted parent ions in the absence of laser irradiation, which would otherwise manifest itself in the IRMPD spectra as a baseline offset. An IRMPD spectrum was produced by plotting the photodissociation yield (PDY) with a 3-point moving average filter as a function of laser frequency. 

During the IRMPD measurements of the dimers, when the laser was off, the monomer peak at the *m/z* of 133 for HAsn^+^ was still observed, whereas no further fragmentation was detected for all the four dimer species. Therefore, the detected monomer fragment peak at the *m/z* of 133 contains contributions from both the laser induced and the self-dissociated fragmentation of the binary amino acid complex. The contribution of the latter can be easily corrected using the usual background subtraction procedure [75]. The detailed procedure is provided in Point S2, Supplementary Material.

### 3.3. Computational Details

The conformational searches were done using the CREST program [38]. The low- level geometry optimization and single-point energy calculations were carried out using the Molpro software package [48,49]. The final full geometry optimization and harmonic frequency calculations were carried out using Gaussian software packages [50]. A flow chart of the three-tiered approach used in the current study including the software packages used, the levels of theory, and the example input files is provided in Point S3, Supplementary Material. All harmonic wavenumber axes were scaled by a factor of 1.04 for easy comparison of the experimental spectra with their harmonically predicted counterparts. The Cartesian coordinates of the isomers in Figure 6 and Figure 7 are summarized in a zip file and provided in the Supplementary Material.

## 4. Conclusions

In this paper, we report the structural preferences for four homochiral and heterochiral HSerAsn^+^ and HValAsn^+^ dimers investigated using IRMPD spectroscopy, aided by a three-tiered computational approach which explores the conformational spaces of the four dimers systematically. The conformational space found for HSerAsn^+^ and HValAsn^+^ dimers are classified into Type I, II, III and ZW, based on their binding topologies. The main species responsible for the experimentally observed IRMPD spectra are identified as Type I structures, where serine or valine form strong intermolecular hydrogen bonds from the NH_3_^+^ group to the carbonyl O and NH_2_ of asparagine, despite the fact that Type II structures of *R*,*S*-HSerAsn^+^ are predicted to be more stable than its Type I structures. In the case of HSerAsn^+^, Type III structures also make some contribution. This was explained based on a partially kinetically controlled dimer formation process where the abundance of the protonated amino acid subunit plays a role in the final abundance of the corresponding dimer. We note that the protonated subunits in the observed dimers correspond to the most stable isomers of the isolated protonated amino acids detected experimentally in several previous IRMPD studies. A free energy gap of 2.3 kJ mol^−1^ was predicted between the experimentally observed homo- and heterochiral HSerAsn^+^ dimers, while that value drops to 0.7 kJ mol^−1^ between the observed *S*,*S*- and *R*,*S*-HValAsn^+^ dimers. The corresponding ZPE corrected energy gaps are 8.3 and 1.7 kJ mol^−1^ for *S*,*S*- versus *R*,*S*-HSerAsn^+^ and *S*,*S*- versus *R*,*S*-HValAsn^+^, respectively, suggesting that a cryogenic experimental temperature may better tease out the chirality recognition spectral signatures in these systems. 

## Figures and Tables

**Table 1:** The monomeric composition of the lowest energy isomers of each type for the four binary species studied.

Isomer Ranking ^a^	*S*,*S*-HSerAsn^+^	*R*,*S*-HSerAsn^+^	*S*,*S*-HValAsn^+^	*R*,*S*-HValAsn^+^
	Type, Ser/Asn Comp ^b^	Type, Ser/Asn Comp ^b^	Type, Val/Asn Comp ^b^	Type, Val/Asn Comp ^b^
1	Type ZW1, HS1^+^/ZW	Type ZW1, HS1^+^/ZW	Type I, HV1^+^/A1 ^c^	Type I, HV1^+^/A1 ^c^
2	Type I, HS1^+^/A1 ^c^	Type II, HS3^+^/A2	Type ZW1, HV1^+^/ZW	Type ZW1, HV1^+^/ZW
3	Type II, HS1^+^/A2	Type I, HS1^+^/A1^c^	Type III, HV1^+^/A2	Type III, HV1^+^/A2
4	Type III, S1/HA^+ c^	Type III, S1/HA^+c^	Type II, HV3^+^/A2	Type II, HV3^+^/A2

^a^ Based on ∆G with respect to the most stable isomer of each type. Note that the ordering of Type I and Type II of *S*,*S*- and *R*,*S*-HSerAsn^+^ is reversed. ^b^ The composition of the two monomeric subunits. S = Ser; V = Val; A = Asn. We add “H” and “+” for the protonated species and 1, 2, etc., indicates the relative stability of the protonated subunit going from the most stable one to the least. For the neutral subunits, 1, 2, etc., are used to indicate the structural difference among different subunits, rather than energy ordering, because the neutral monomeric structure inside a dimer species often differs greatly from its isolated neutral ones. See the main text for discussions. ^c^ The shaded ones are those assigned, i.e., observed experimentally, in Figure 6 and Figure 7.

## Data Availability

Data is contained within the article.

## Supplementary Materials

The following are available online. Tables S1–S4: Relative energies of the isomer of homo- and heterochiral HSerAsn^+^ and HValAsn^+^; Table S5: Monomeric compositions of the binary isomers; Figures S1–S4: Comparison of theoretical and experimental IR spectra of homo- and heterochiral HSerAsn^+^ and HValAsn^+^; Figure S5: Predicted IR spectra in the 0–2650 cm^−1^ region of the observed HSerAsn^+^ and HValAsn^+^ isomers; Figure S6: NCI plots of the most stable homo- and heterochiral HValAsn^+^ isomers; Point S1: Instrumentation modification; Point S2: Photodissociation yield calculations; Point S3: Computational details; An xyz.zip file: Cartesian coordinates of the isomers, for which IR spectra are shown in Figure 6 and Figure 7.

## Sample Availability

Samples of the compounds are available from the authors.
