# Supplementary material for: Vibrational Spectroscopy of Homo- and Heterochiral Amino Acid Dimers: Conformational Landscapes
Source: Molecules. 2021 Dec 22;27(1):38. doi: 10.3390/molecules27010038 (PMC8746356; doi:10.3390/molecules27010038)
Supplement: Supplementary file 1 [file molecules-27-00038-s001.zip › ESI-Molecules-Dec20.pdf]

molecules

Supplementary Material

Vibrational Spectroscopy of Homo- and Heterochiral Amino Acid Dimers: Conformational Landscapes

Haolu Wang,[1] Matthias Heger,[1] Mohamad H. Al-Jabiri,[1] and Yunjie Xu[1,*]

[1] Department of Chemistry, University of Alberta, Edmonton, Alberta, Canada; haolu@ualberta.ca; heger@ualberta.ca; aljabiri@ualberta.ca

* Correspondance: yunjie.xu@ualberta.ca; Tel.: 1-780-492-1244.

Contents

Table S1-S4. Relative energies of the homo- and heterochiral $HSerAsn^+$ and $HValAsn^+$ isomers within 10 kJ mol^{-1} .. S2-3

Table S5. Monomeric compositions of all isomers within 10 kJ mol^{-1}.......... S4

Figure S1-S4. Comparison of theoretical and experimental IR spectra of $HSerAsn^+$ and $HValAsn^+$ isomers.......... S5-7

Figure S5. Predicted IR spectra of the species assigned in the 0-2650 cm^{-1} region S8

Figure S6. NCI plots of the most stable homo- and heterochiral $HValAsn^+$ isomers.......... S9

Point S1. Instrumentation modifications.......... S9-10

Point S2. Photo-dissociation yield calculations S10-11

Point S3. Computational details S11-12

Table S1. Calculated relative ZPE corrected electronic energies, (ΔE) and Gibbs free energies, (ΔG) in kJ mol^{-1} of the *S,S*-HSerAsn$^+$ isomers and the percentage Boltzmann factor (B_f, %) based on ΔG at room temperature.

Isomer#[a]	Type[b]	ΔE[c]	ΔE(PCM)[d]	ΔG[c]	B_f
1	ZW	3.7	13.7	0	36.7
2	I	0	28.9	2.5	13.2
3	II	0.7	20.5	3.3	9.7
4	ZW	1.9	1.8	3.4	9.4
5	III	4.0	29.2	4.3	6.5
6	II	2.3	21.7	4.7	5.6
7	OTHER	6.2	32.4	5.6	3.8
8	I	7.2	30.7	6.1	3.1
9	III	4.2	25.5	6.2	3.0
10	ZW	5.6	0	6.4	2.8
11	OTHER	1.2	25.9	6.8	2.4
12	I	6.4	33.2	7.7	1.7
13	OTHER	3.3	30.2	8.7	1.1
14	OTHER	5.3	18.2	9.1	1.0

[a] The ranking is based on ΔG, with 1 being the most stable one. The shaded ones are those observed experimentally. See the main text for discussions.
[b] See the main text for the definition and discussion of different types.
[c] Calculated at the B3LYP-D3BJ/def2-TZVP level using G16.
[d] Single point energies at the B3LYP-D3BJ/def2-TZVP level with the inclusion of the 1:1 mixed water + methanol solvent using the PCM model with a dielectric constant of 55.48415.

Table S2. Calculated relative ZPE corrected electronic energies, (ΔE) and Gibbs free energies, (ΔG) in kJ mol^{-1} of the *R,S*-HSerAsn$^+$ isomers and the percentage Boltzmann factor (B_f, %) based on ΔG at room temperature.

Isomer#[a]	Type[b]	ΔE[c]	ΔE(PCM)[d]	ΔG[c]	B_f
1	ZW	4.0	14.9	0	38.1
2	ZW	1.9	3.0	3.2	10.5
3	II	0	20.0	3.7	8.5
4	ZW	4.1	0	4.4	6.5
5	I	8.3	32.2	4.5	6.1
6	III	8.7	33.9	4.6	6.0
7	I	6.0	34.6	4.7	5.8
8	II	2.9	23.1	4.8	5.5
9	OTHER	6.7	34.4	5.2	4.8
10	I	4.3	31.9	6.2	3.1
11	OTHER	9.8	32.5	7.3	2.0
12	III	8.8	30.5	8.4	1.3
13	OTHER	4.5	17.1	8.8	1.1
14	OTHER	6.4	22.0	9.4	0.8

[a] The ranking is based on ΔG, with 1 being the most stable one. The shaded ones are those observed experimentally. See the main text for discussions.
[b] See the main text for the definition and discussion of different types.
[c] Calculated at the B3LYP-D3BJ/def2-TZVP level using G16.
[d] Single point energies at the B3LYP-D3BJ/def2-TZVP level with the inclusion of the 1:1 mixed water + methanol solvent using the PCM model with a dielectric constant of 55.48415.

Table S3. Calculated relative ZPE corrected electronic energies, (ΔE) and Gibbs free energies, (ΔG) in kJ mol^{-1} of the *S,S*-$HValAsn^{+}$ isomers and the percentage Boltzmann factor (B_f, %) based on ΔG at room temperature.

Isomer#[a]	Type[b]	ΔE[c]	ΔE(PCM)[d]	ΔG[c]	B_f
1	I	0	14.7	0	33.6
2	ZW	3.3	0	1.6	17.6
3	I	0.3	13.6	1.9	15.6
4	III	2.7	14.6	2.2	13.6
5	I	0.6	13.8	2.6	11.9
6	OTHER	6.0	20.0	6.0	3.0
7	OTHER	6.0	22.7	6.2	2.7
8	II	2.7	15.5	7.2	1.9

[a] The ranking is based on ΔG, with 1 being the most stable one. The shaded ones are those observed experimentally. See the main text for discussions.
[b] See the main text for the definition and discussion of different types.
[c] Calculated at the B3LYP-D3BJ/def2-TZVP level using G16.
[d] Single point energies at the B3LYP-D3BJ/def2-TZVP level with the inclusion of the 1:1 mixed water + methanol solvent using the PCM model with a dielectric constant of 55.48415.

Table S4. Calculated relative ZPE corrected electronic energies, (ΔE) and Gibbs free energies, (ΔG) in kJ mol^{-1} of the *R,S*-$HValAsn^{+}$ isomers and the percentage Boltzmann factor (B_f, %) based on ΔG at room temperature.

Isomer#[a]	Type[b]	ΔE[c]	ΔE(PCM)[d]	ΔG[c]	B_f
1	I	2.0	21.2	0	32.8
2	I	0	16.7	0.1	31.8
3	ZW	3.5	0	1.3	19.4
4	III	2.9	16.8	3.2	9.0
5	I	5.9	16.3	4.8	4.7
6	II	3.9	14.7	7.9	1.3
7	OTHER	7.4	20.9	9.0	0.9

[a] The ranking is based on ΔG, with 1 being the most stable one. The shaded ones are those observed experimentally. See the main text for discussions.
[b] See the main text for the definition and discussion of different types.
[c] Calculated at the B3LYP-D3BJ/def2-TZVP level using G16.
[d] Single point energies at the B3LYP-D3BJ/def2-TZVP level with the inclusion of the 1:1 mixed water + methanol solvent using the PCM model with a dielectric constant of 55.48415.

Table S5. The monomeric composition of all isomers within 10 kJ mol^{-1} for the four binary species studied.

Isomer ranking[a]	*S,S*-HSerAsn$^+$	*R,S*-HSerAsn$^+$	*S,S*-HValAsn$^+$	*R,S*-HValAsn$^+$
	Type, Ser/Asn Comp[b]	Type, Ser/Asn Comp[b]	Type, Val/Asn Comp[b]	Type, Val/Asn Comp[b]
1	Type ZW1, HS1$^+$/ZW	Type ZW1, HS1$^+$/ZW	Type I, HV1$^+$/A1[c]	Type I, HV1$^+$/A1[c]
2	Type I, HS1$^+$/A1[c]	Type ZW2, HS3$^+$/ZW	Type ZW1, HV1$^+$/ZW	Type I, HV1$^+$/A1
3	Type II, HS1$^+$/A2	Type II, HS3$^+$/A2	Type I, HV1$^+$/A1	Type ZW1, HV1$^+$/ZW
4	Type ZW2, HS1$^+$/ZW	Type ZW2, HS2$^+$/ZW	Type III, HV1$^+$/A2	Type III, HV1$^+$/A2
5	Type III, S1/HA$^+$[c]	Type I, HS1$^+$/A1[c]	Type I, HV1$^+$/A1	Type I, HV1$^+$/A1
6	Type II, HS2$^+$/A2	Type III, S1/HA$^+$[c]	OTHER, HV1$^+$/A2	Type II, HV3$^+$/A2
7	OTHER, HS1$^+$/A2	Type I, HS1$^+$/A1	OTHER, HV1$^+$/A1	OTHER, HV1$^+$/A2
8	Type I, HS1$^+$/A1	Type II, HS2$^+$/A2	Type II, HV3$^+$/A2	
9	Type III, S1/HA$^+$	OTHER, HS1$^+$/A2		
10	Type ZW2, HS1$^+$/ZW	Type I, HS1$^+$/A1		
11	OTHER, HS3$^+$/A2	OTHER, HS1$^+$/A2		
12	Type I, HS3$^+$/A1	Type III, S1/HA$^+$		
13	OTHER, HS2$^+$/A2	OTHER, HS1$^+$/A1		
14	OTHER, HS1$^+$/A1	OTHER, HS1$^+$/A2		

[a] Ordering based on ΔG with respective to the most stable isomer of each type. Note the ranking of Type I and II of *S,S*- and *R,S*-HSerAsn$^+$ is reversed.

[b] The composition of the two monomeric subunits. S = Ser; V= Val; A = Asn; ZW = zwitterionic form. We add "H" and "+" for the protonated species and 1, 2, etc, indicates the relative stability of the protonated subunit, with "1" being the most stable one. For the neutral subunits, 1, 2, etc, are used to indicate the structural difference among different subunits, rather than energy ordering because the associated structure inside a dimer species often differs greatly from its isolated neutral ones. See the main text for discussions.

[c] The shaded ones are those assigned, i.e. observed experimentally in Figure 6 and 7.

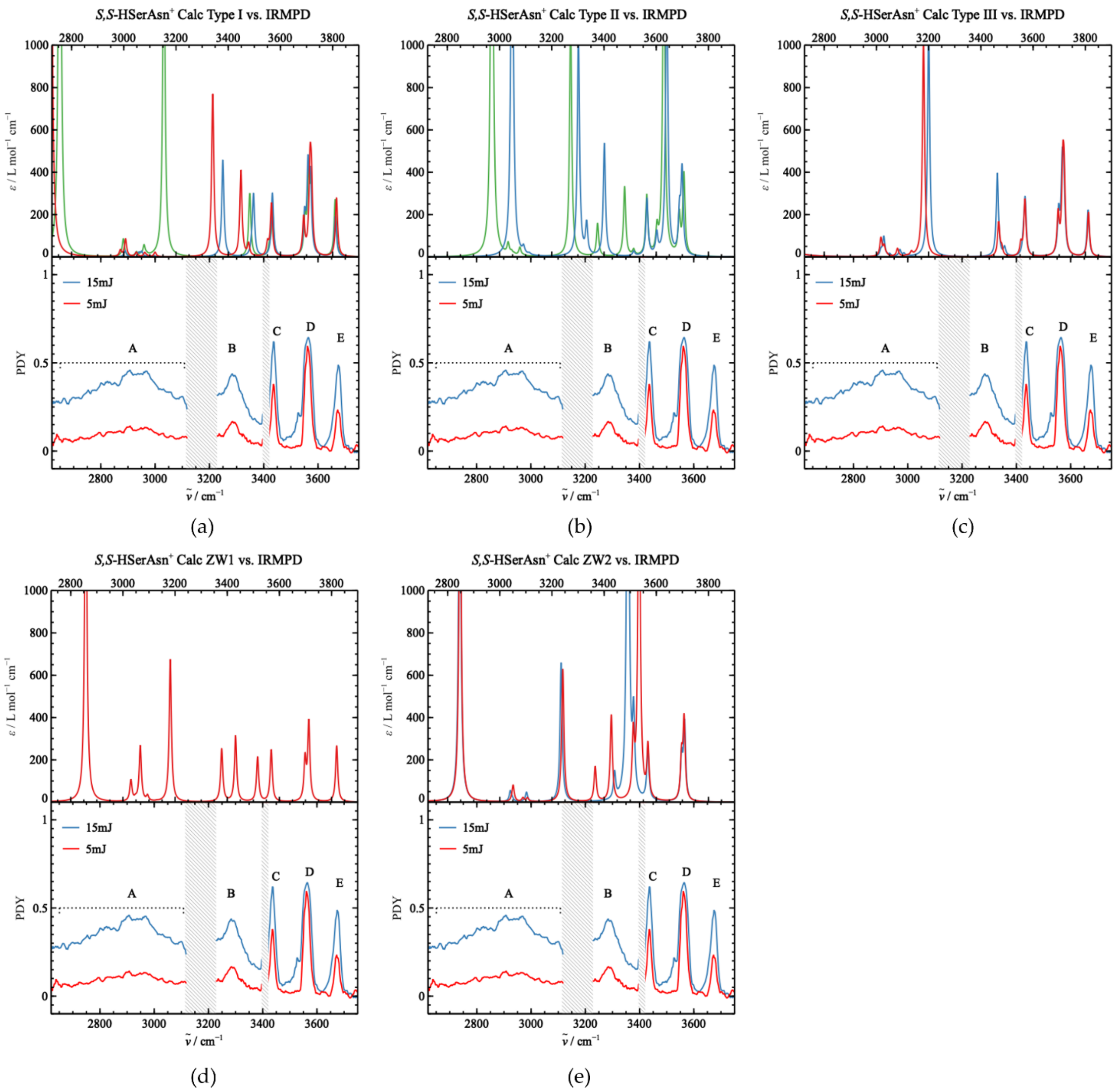

Figure S1. Comparison of the experimental IR spectra of *S,S*-HSerAsn+ with the combined theoretical IR spectra of all dimer structures in (a) Type I, (b) Type II, (c) Type III, (d)Type ZW1, and (e) Type ZW2.

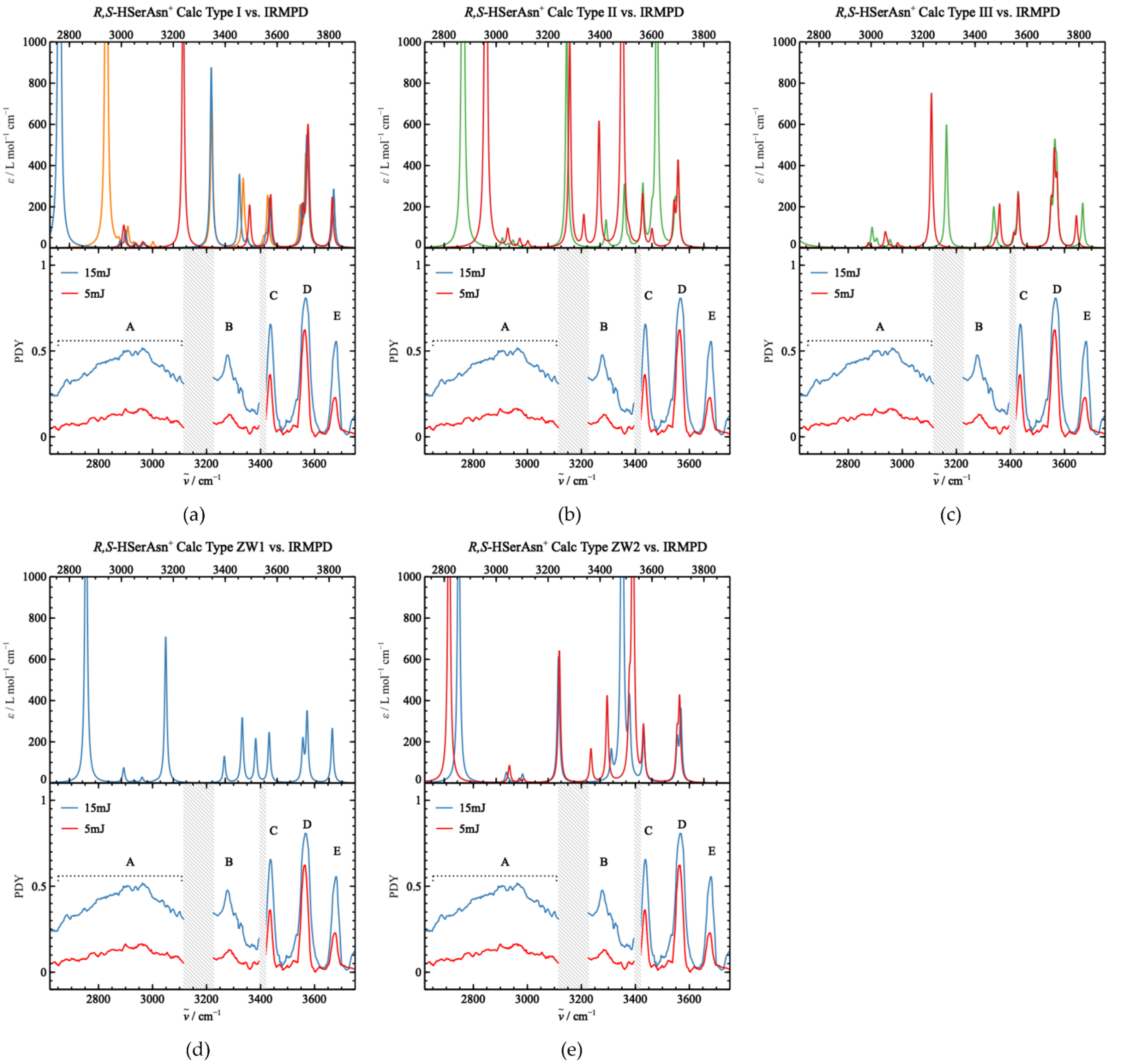

Figure S2. Comparison of the experimental IR spectra of *R,S*-HSerAsn+ with the combined theoretical IR spectra of all dimer structures in (a) Type I, (b) Type II, (c) Type III, (d)Type ZW1, and (e) Type ZW2.

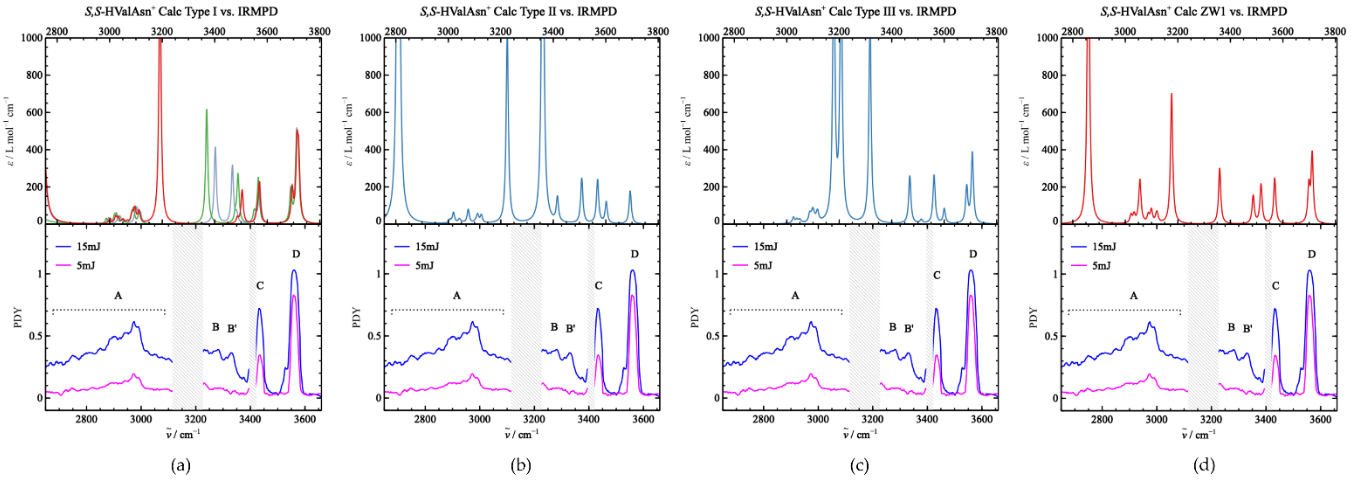

Figure S3. Comparison of the experimental IR spectra of *S,S*-HValAsn^{+} with the combined theoretical IR spectra of all dimer structures in (a) Type I, (b) Type II, (c) Type III, and (d)Type ZW1.

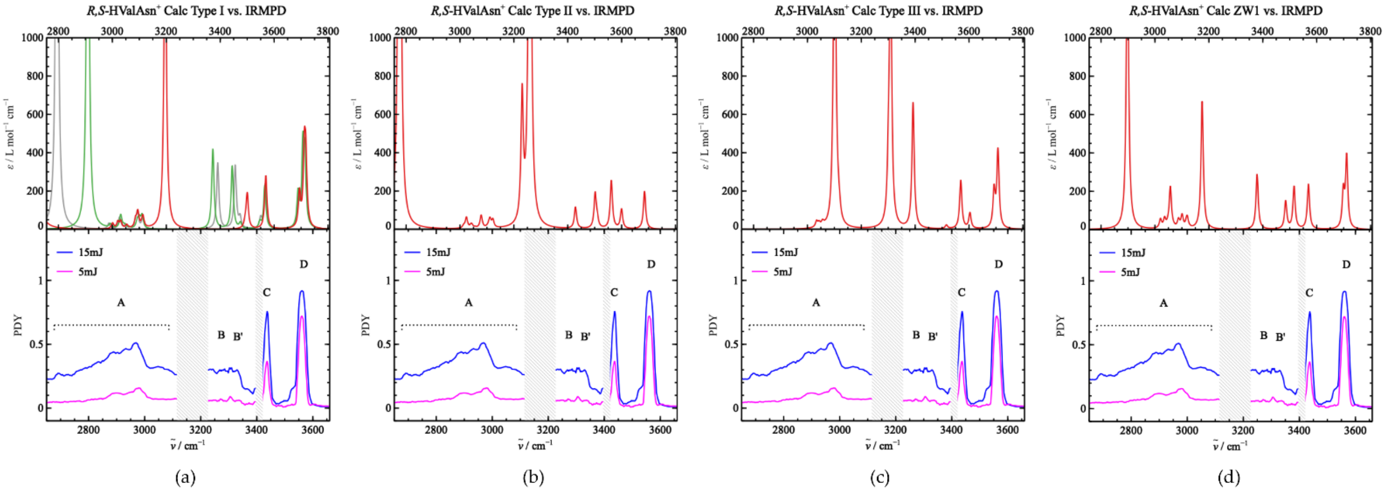

Figure S4. Comparison of the experimental IR spectra of *R,S*-HValAsn^{+} with the combined theoretical IR spectra of all dimer structures in (a) Type I, (b) Type II, (c) Type III, and (d)Type ZW1.

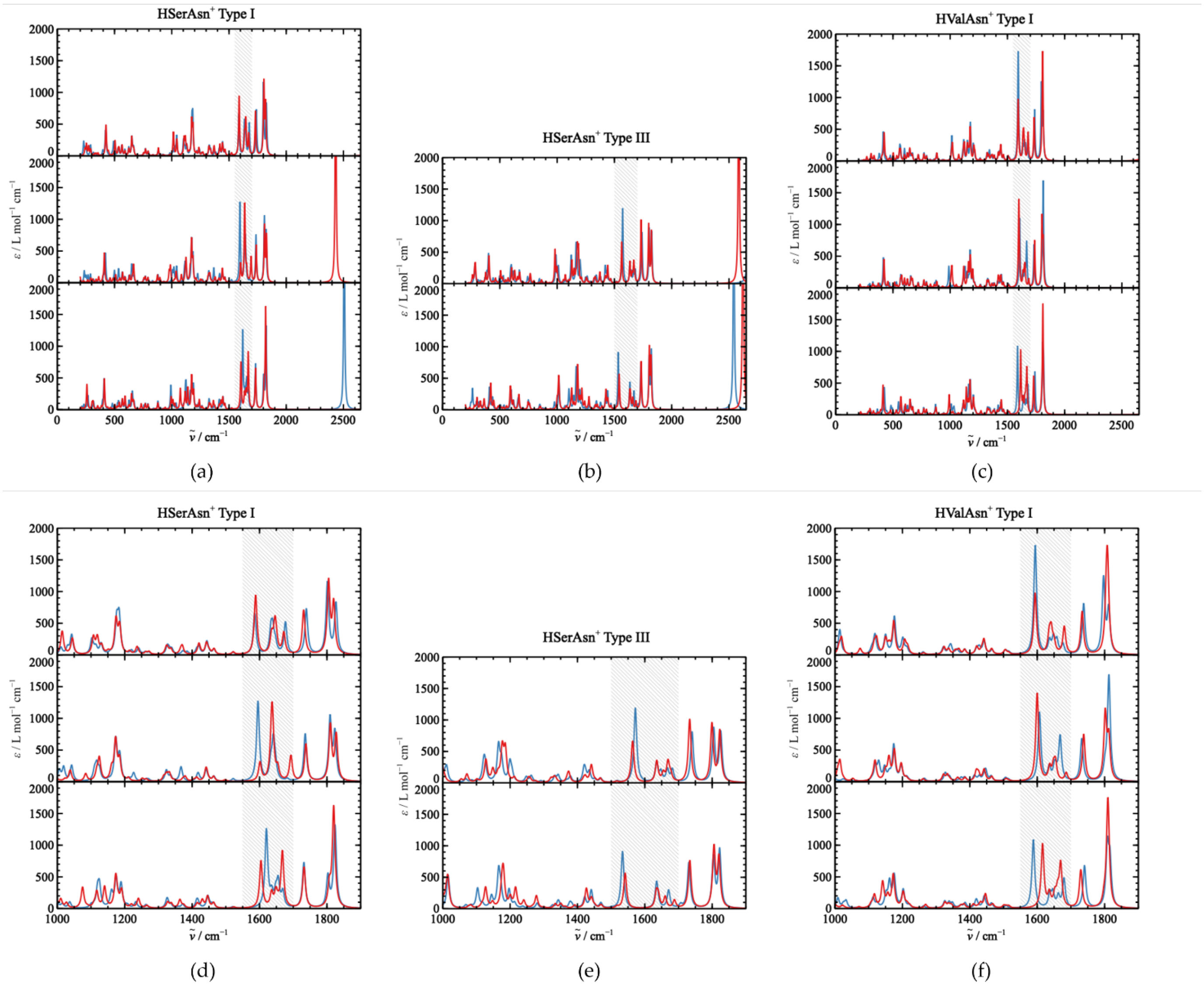

Figure S5. Predicted IR spectra in the 0-2650 cm^{-1} region of (a) $HSerAsn^+$ Type I structures, (b) $HSerAsn^+$ Type III structures, and (c) $HValAsn^+$ Type I structures. IR spectra of the homochiral binary species are shown in the red color, whereas those of the heterochiral species are shown in the blue color blue. The corresponding zoom-in spectra in the 1000-1900 cm^{-1} region are given in (d), (e) and (f). The “shaded” area indicates the region where chirality recognition differences between the homo- and heterochiral species are most prominent, where the absorption bands are associated with the scissoring or twisting motions of the NH_3^+ and the sidechain NH_2 functional groups, as well as the NH_3 umbrella bending motion.

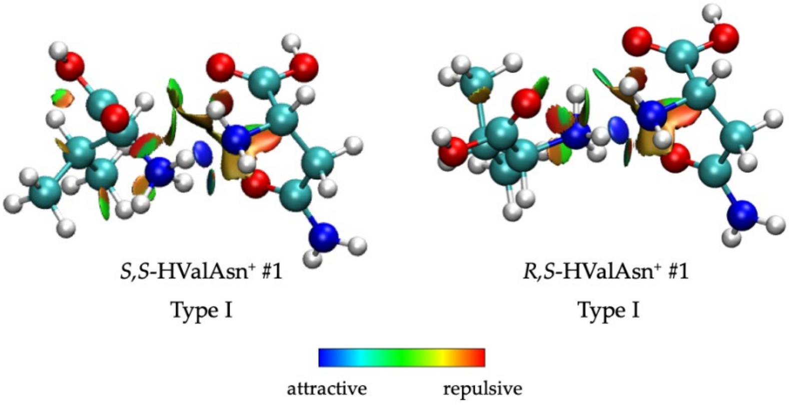

Figure S6. NCI plots of the most stable homo- and heterochiral $HValAsn^+$ at an iso-surface value of 0.5.

Point S1: Instrumentation Modifications

Compared to the previously reported "SCORPION" instrument [1], a range of modifications were made. The main details are provided below.

Ion Trapping Cycle: The IRMPD experiment is based around a repeated event cycle. First, the Paul trap (manufactured by Jordan TOF Products, Inc.) is loaded with ions for a set amount of time. Second, the ion influx is blocked off and the trap is kept "isolated" to allow for thermalization and irradiation. Third, the trap content is ejected and guided to the TOF for analysis. The power supply that drives the Paul trap accepts an external analog signal to scale the peak-to-peak RF voltage on the trap. We use an Agilent 33220 function generator with an arbitrary waveform module to produce a custom voltage profile on the trap, similar to the scheme previously described by Gulyuz et al. [2]. In this voltage profile, the trapping RF potential is kept low while the trap is being filled; then it is ramped up temporarily to a high voltage, before dropping back to an intermediate voltage level for the remainder of the trapping cycle. The idea is to eject any unwanted fragments that might have been produced while filling the ion trap under its raised helium pressure conditions. We name these voltage curves "ADSR profiles" due to their similarity to the envelope curves of the same name found in audio engineering. The last section of the ion guide that feeds into the Paul trap is outfitted with field-shaping "asymptote" electrodes and can itself be fed with a stream of helium buffer gas to act as, for example "pre-trapping", after some modifications to the helium feed and vacuum system. One can now store the ions that are continuously produced by the ESI source while the Paul trap is in its isolated trapping period. These stored ions are then injected into the trap upon the next measurement cycle instead of being lost, greatly enhancing the overall ion abundance.

Laser Radiation: Previously, the trap content was irradiated during the entire measurement cycle. In the current experiment, a remote-controlled laser shutter (Vincent Associates TS-6B, later replaced with an ES-6B unit) was introduced to control the nominal irradiation energy based on the cw laser power and shutter opening times. This helps to avoid saturation of the IRMPD spectra due to full depletion of the parent ion intensities. Since a cw laser is used, an energy value per shutter opening time for the spectra, for example 5 mJ and 15 mJ, is given, instead of a laser power reading. In the current dimer experiments, we found that the fragmentation yield into monomer units was more or less linear with the nominal irradiation energy. Thus, the shutter opening time is adjusted to give the same nominal irradiation energy for each scan.

System Interfacing and Data Treatment: To conveniently control different components of the instrument, a custom GUI-driven software suite was created in Python. The software takes control of the ion optics power supplies, pulse generators for the synchronization of timed events, and various devices required for remotely steering the Argos OPO laser

[3]. It also includes a tool to record data from the TOF in a customizable manner that is specifically tailored to our data analysis strategy. With the helium buffer gas injected into the pre-trapping ion guide and the Paul trap, these sections of the instrument can essentially act as collision-induced dissociation cells for the incoming ions. This may lead to the appearance of fragment peaks in the mass spectra, causing a non-zero photo-dissociation yield (PDY) even without laser irradiation. Even with extensive retuning of the ion optics and implementation of the ADSR ion trapping profiles described above, this background fragmentation was impossible to be fully suppressed for the weakly bound dimers studied in this paper. The integration of the external laser shutter described above, however, proves to be useful in this regard as well. A Berkeley Nucleonics Model 555 pulse generator is used to trig the laser shutter on every second measurement cycle and the irradiated and the non-irradiated (background) spectra of the trapped ions are recorded. A custom Python script is used to analyze the recorded mass spectra where the irradiated and non-irradiated measurement cycles are treated separately, allowing background subtraction in the final IRMPD spectrum.

---

1 Heger, M.; Cheramy, J.; Xie, F.; Chen, Z.; Xu. Y. *J. Mol. Spectroc.* **2018**, *352*, 36-44.

2 Gulyuz, K.; Stedwell, C. N.; Wang, D.; Polfer, N. C. *Rev. ci. Instrum.* **2011**, *82*, 54101.

3 Morrison, A. M.; liang, T.; Douberly, G. E.; *Rev. Sci. Instrum.* **2013**, *84*, 13102.

Point S2: Photo-Dissociation Yield Calculation

An IRMPD spectrum can be generated by plotting the photo-dissociation yield (PDY) at each laser frequency using the following equation:

$$PDY = \frac{\Sigma_i I_i}{I_p + \Sigma_i I_i} \tag{1}$$

where I_p is the intensity of the molecular ion after dissociation and I_i is the detected intensity of the ionic fragment *i*. Since we have one main monomeric fragment, $HAsn^+$ (m/z=133), we can simply it to the contribution of each individual fragment ion is represented by:

$$PDY_i = \frac{I_i}{I_p + \Sigma_i I_i} \tag{2}$$

where with $\Sigma_i\ PDY_i = PDY$. Because of its non-covalently bonding nature, the background dissociation discussed in Point S1 results in non-zero PDY in the monomer mass channels even without laser irradiation. This can be easily corrected by taking the difference of the irradiated (PDY_i) and background (PDY_i^0) scan to obtain the corrected $\overline{PDY_i}$:

$$\overline{PDY_i} = PDY_i - PDY_i^0 \tag{3}$$

Only one monomeric fragment, $HAsn^+$ (m/z=133), is observed in the current experiments. By monitoring its intensity, I_{133}, and using equation (3), an IRMPD spectrum (red in Figure S7) is generated. There is a negative band at 3510 cm^{-1}, indicating that $_{133}$, is higher with laser is off than with laser on. This can happen because with laser on, $HAsn^+$ can further dissociate into smaller fragments by losing H_2O and H_2O + CO_2, thus generating m/z peak at 115 and 87, respectively. By adding up I_{87} and I_{115} to I_{133}, one obtains $I_{133,sum}$ which is the amount of $HAsn^+$ with laser on and without further fragmentation. Since we observed no evidence of further fragmentation of $HAsn^+$ with laser off, using equation (3) with $I_{133,sum}$, the proper IRMPD spectrum is obtained (the blue trace in Figure S7), with a small positive peak at 3510 cm^{-1}, the same one presented in the main text.

.

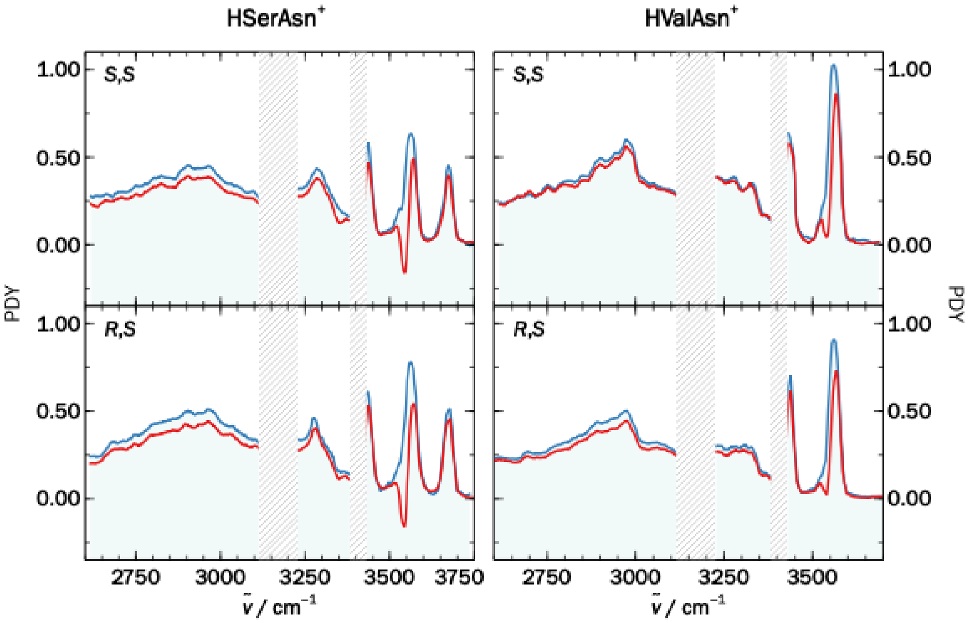

Figure S7. IRMPD spectra at 15 mJ generated by using the $HAsn^{+}$ intensity directly (the red trace) and by taking into account the further fragmentation of $HAsn^{+}$ (the blue trace). Please see the text for details. The two laser "blind" regions mentioned in the experimental section are indicated by the shaded areas.

Point S3: Computational Details

A flow chart of the three-tiered approach is illustrated below:

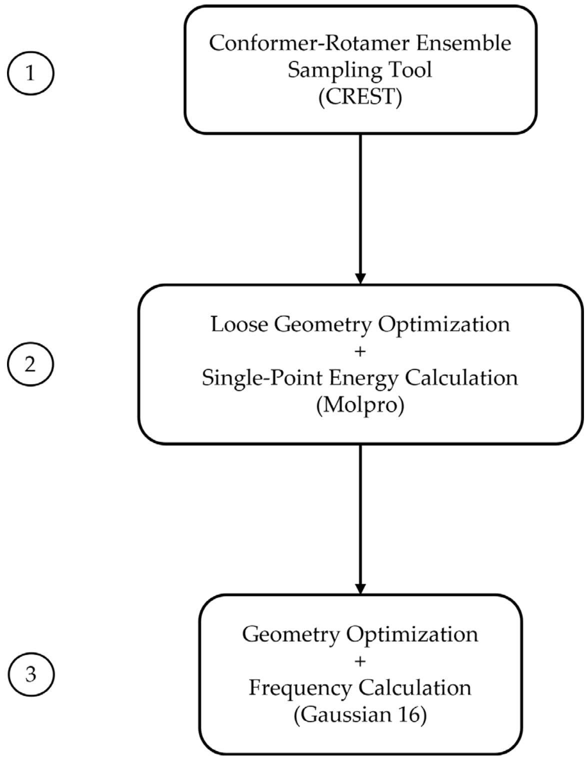

1. We ran CREST multiple times with the same and different starting geometries including both *cis-* and *trans-*COOH configurations of the three amino acid subunits. To remove the redundant structures, we used a Python program to calculate the root-mean-square deviation (RMSD) of atomic Cartesian coordinates for the obtained conformations. A

RMSD value of zero indicates identical conformations, whereas the larger the value, the more unalike the two conformations are. By trial and error, a RMSD threshold of 0.6 was used in the current study. The RMDS program written in Python can be obtained from the authors upon request.

2. The key word line used in the Molpro loose optimization and single point energy calculations is provided below:

revPBE-D3(0)/def2-SVP loose opt + B3LYP-D3(0)/def2-TZVP single point

Again, we applied the RMSD program to remove redundant structures at this step.

3. We selected all isomers within an energy window of 10 kJ mol^{-1} using the single point energies obtained above. In the final step, we carried out full geometry optimization and harmonic frequency calculations at the B3LYP-D3BJ/def2-TZVP level using Gaussian 16. The key word lines are provided below:

```
{%chk=}
# opt b3lyp def2tzvp empiricaldispersion=gd3bj freq

{title card}

{charge, multiplicity}
{molecule specification}
```

In addition, we also carried out single point energy calculations with the inclusion of the mixed solvent using the PCM implemented in Gaussian 16. The key word lines are provided below:

```
{%chk=}
# sp b3lyp def2tzvp empiricaldispersion=gd3bj SCRF=(Solvent=Generic, Read)

{title card}

{charge, multiplicity}
{molecule specification}

EPS={add the value of linear combination of both solvents}
EpsInf=(add the value of linear combination of both solvents)
```

The dielectric constant of a binary solvent is calculated using the formula below:

$$\epsilon_m = x_1\epsilon_1 + x_2\epsilon_2$$

where x_1 and x_2 are the mole fractions of solvent 1 and 2, respectively, in the binary mixture, and ϵ_1 and ϵ_2 are their correpsonding dielectric constants. The dielectric constants of water and methanol are 78.3553 and 32.613, respectively. The dielectric constant of the 1:1 water + methanol solvent is ϵ=0.5(78.3553)+0.5(32.613) = 55.48415.
